# Supplementary material for: Mechanism of glycoform specificity and in vivo protection by an anti-afucosylated IgG nanobody
Source: Nat Commun. 2023 May 18;14:2853. doi: 10.1038/s41467-023-38453-1 (PMC10195009; doi:10.1038/s41467-023-38453-1)
Supplement: Supplementary file 1 — Supporting Information [file 41467_2023_38453_MOESM1_ESM.pdf]

Supplementary Information

Mechanism of glycoform specificity and *in vivo* protection by an anti-afucosylated IgG nanobody

Aaron Gupta[1]†, Kevin Kao[1]†, Rachel Yamin[1], Deena A. Oren[2], Yehuda Goldgur[3], Jonathan Du[4], Pete Lollar[5], Eric J. Sundberg[4], Jeffrey V. Ravetch[1]*

[1] Laboratory of Molecular Genetics & Immunology, The Rockefeller University, New York, NY, USA.

[2] Structural Biology Resource Center, The Rockefeller University, New York, NY, USA.

[3] Structural Biology Program, Memorial Sloan Kettering Cancer Center, New York, NY, USA.

[4] Department of Biochemistry, Emory University School of Medicine, Atlanta, GA, USA.

[5] Department of Pediatrics, Emory University School of Medicine, Atlanta, GA, USA.

†These authors contributed equally to this work.

*Corresponding author. Email: ravetch@rockefeller.edu

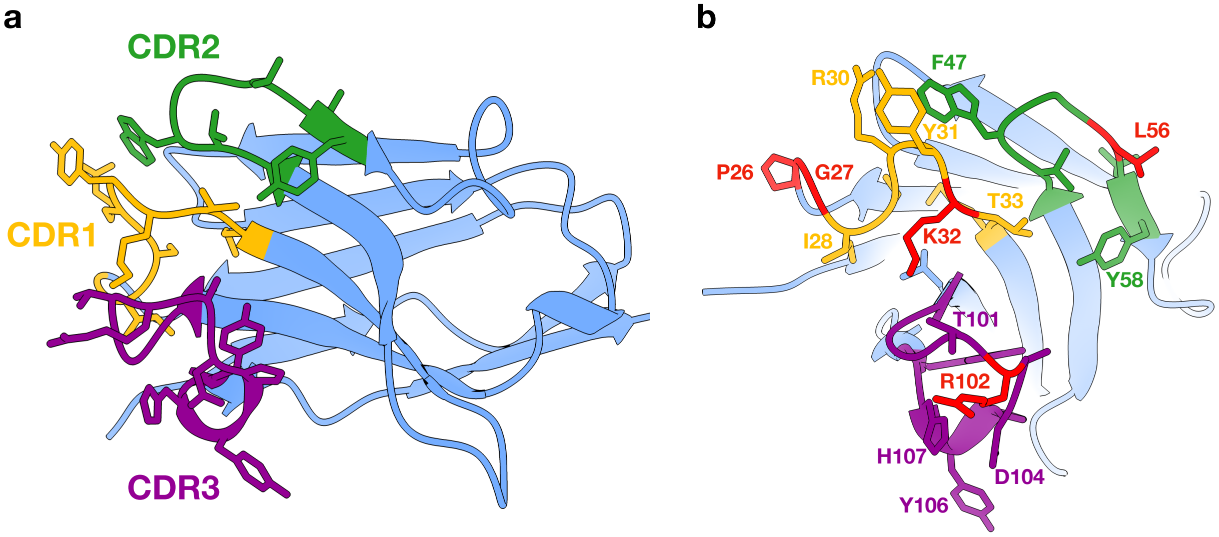

Supplementary Figure 1: Crystal structure of the unbound nanobody X0. a Cartoon representation of the overall structure of unbound X0. Stick model of amino acid sidechains are shown for each CDR loop (CDR1 in gold; CDR2 in forest green; CDR3 in dark purple). Framework regions are shown in cornflower blue. **b** Straight-on view of CDR loops. Residues in red were variable during affinity maturation of C11, while those consistent with CDR coloring in **(a)** were conserved across all affinity-matured clones.

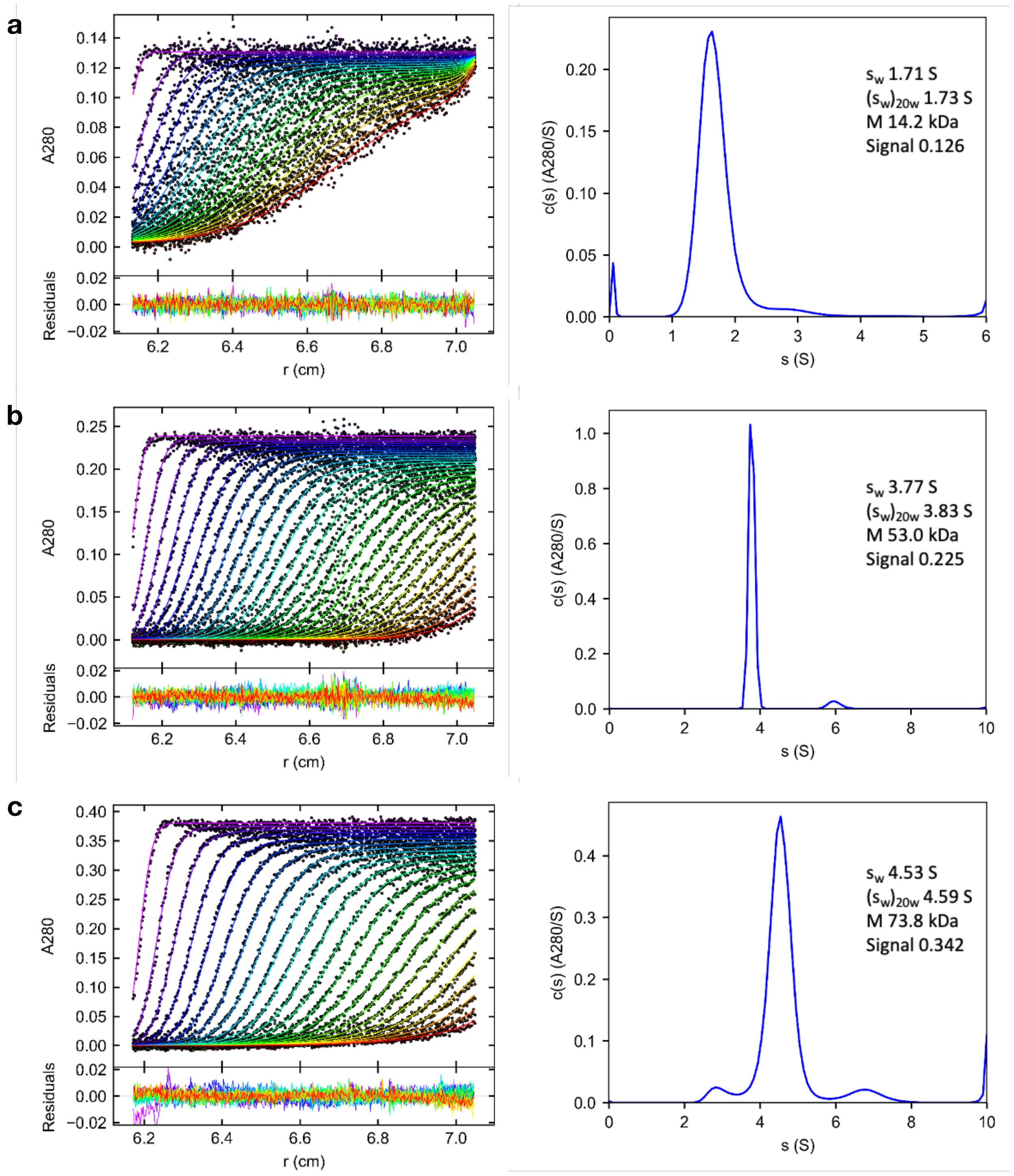

a
A280
Residuals
r (cm)
c(s) (A280/S)
s (S)
s_w 1.71 S
$(s_w)_{20w}$ 1.73 S
M 14.2 kDa
Signal 0.126
b
A280
Residuals
r (cm)
c(s) (A280/S)
s (S)
s_w 3.77 S
$(s_w)_{20w}$ 3.83 S
M 53.0 kDa
Signal 0.225
c
A280
Residuals
r (cm)
c(s) (A280/S)
s (S)
s_w 4.53 S
$(s_w)_{20w}$ 4.59 S
M 73.8 kDa
Signal 0.342

Supplementary Figure 2: Analytical ultracentrifugation of the X0-IgG1 Fc complex. a-c Raw sedimentation profiles (left) of absorbance at 280 nm versus cell radius for X0, afucosylated IgG1 Fc, and X0-afucosylated IgG1 Fc complex and continuous sedimentation coefficient distribution (right), c(S) curve, obtained with a regularization procedure from the data.

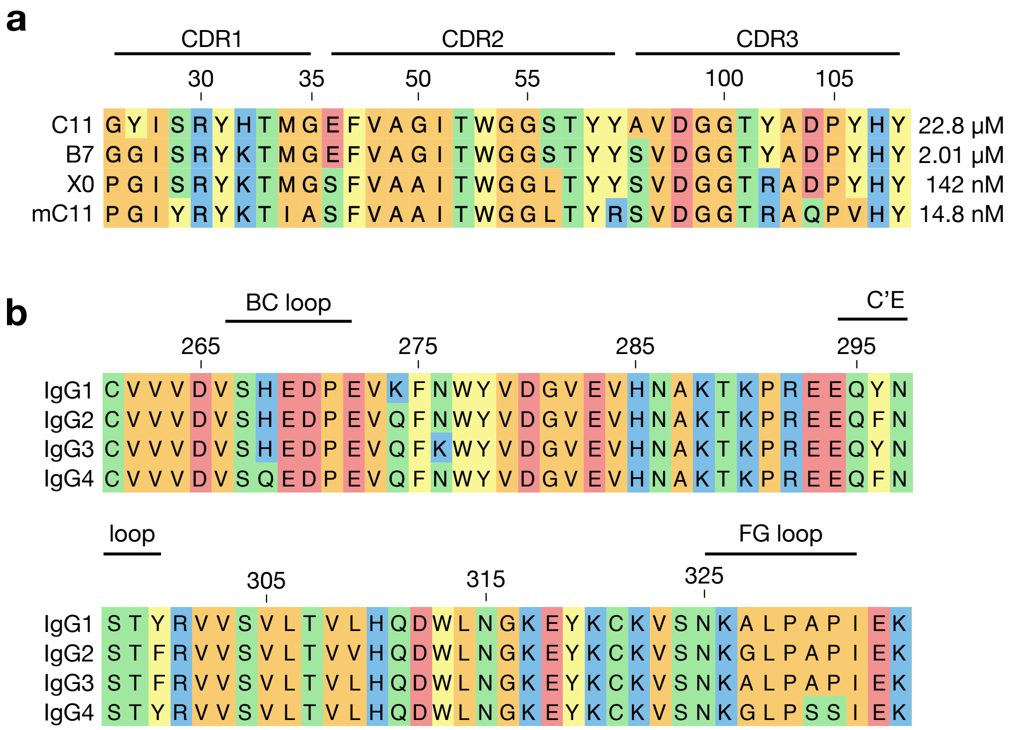

Supplementary Figure 3: Protein sequence alignment of afucosylated IgG-specific nanobody clones from Kao et al. a Clustal Omega alignment of nanobody CDR sequences and associated K_D for afucosylated IgG1. **b** Clustal Omega alignment of IgG1-4 subclass sequences. BC, C’E, and FG loops are noted. Colors indicate amino acid class (hydrophobic in orange, acidic in red, basic in blue, polar in green). Residues are numbered.

a

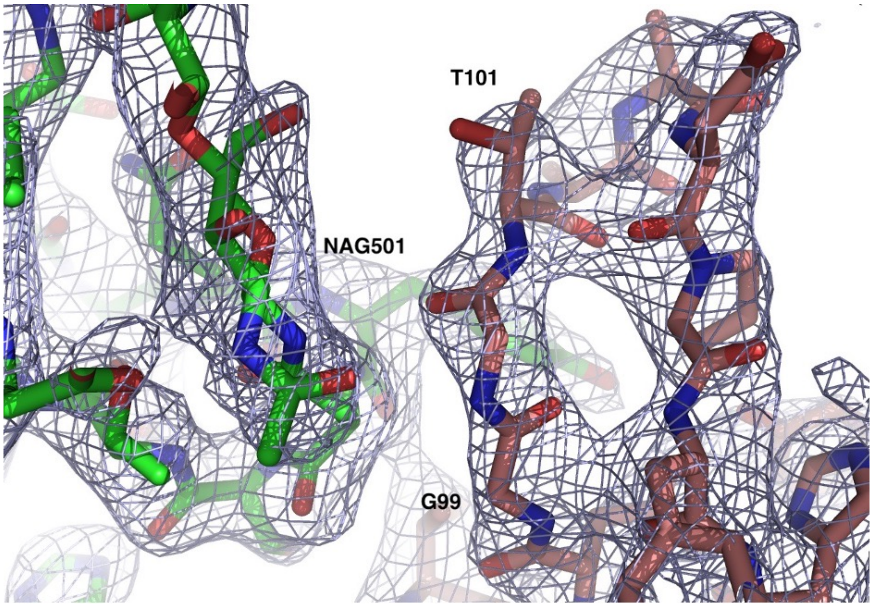

Supplementary Figure 4: Electron density map of X0 CDR3 and the afucosylated IgG1 glycan. a 2Fo − Fc electron density maps contoured at 1.5σ of the binding interface between X0 CDR3 (red) and afucosylated IgG1 glycan (green).
