# Supplementary material for: Mechanism of glycoform specificity and in vivo protection by an anti-afucosylated IgG nanobody
Source: Nat Commun. 2023 May 18;14:2853. doi: 10.1038/s41467-023-38453-1 (PMC10195009; doi:10.1038/s41467-023-38453-1)
Supplement: Supplementary file 3 — Reporting Summary [file 41467_2023_38453_MOESM3_ESM.pdf]

nature portfolio

Corresponding author(s): Jeffrey V. Ravetch

Last updated by author(s): Apr 30, 2023

# Reporting Summary

Nature Portfolio wishes to improve the reproducibility of the work that we publish. This form provides structure for consistency and transparency in reporting. For further information on Nature Portfolio policies, see our Editorial Policies and the Editorial Policy Checklist.

## Statistics

For all statistical analyses, confirm that the following items are present in the figure legend, table legend, main text, or Methods section.

| n/a | Confirmed | |
|---|---|---|
| ☐ | ☒ | The exact sample size (*n*) for each experimental group/condition, given as a discrete number and unit of measurement |
| ☐ | ☒ | A statement on whether measurements were taken from distinct samples or whether the same sample was measured repeatedly |
| ☐ | ☒ | The statistical test(s) used AND whether they are one- or two-sided<br>*Only common tests should be described solely by name; describe more complex techniques in the Methods section.* |
| ☐ | ☒ | A description of all covariates tested |
| ☐ | ☒ | A description of any assumptions or corrections, such as tests of normality and adjustment for multiple comparisons |
| ☐ | ☒ | A full description of the statistical parameters including central tendency (e.g. means) or other basic estimates (e.g. regression coefficient) AND variation (e.g. standard deviation) or associated estimates of uncertainty (e.g. confidence intervals) |
| ☐ | ☒ | For null hypothesis testing, the test statistic (e.g. *F*, *t*, *r*) with confidence intervals, effect sizes, degrees of freedom and *P* value noted<br>*Give P values as exact values whenever suitable.* |
| ☒ | ☐ | For Bayesian analysis, information on the choice of priors and Markov chain Monte Carlo settings |
| ☒ | ☐ | For hierarchical and complex designs, identification of the appropriate level for tests and full reporting of outcomes |
| ☒ | ☐ | Estimates of effect sizes (e.g. Cohen's *d*, Pearson's *r*), indicating how they were calculated |

*Our web collection on statistics for biologists contains articles on many of the points above.*

## Software and code

Policy information about availability of computer code

| | |
|---|---|
| Data collection | Biacore T200 control software v2.0, Microsoft Excel v2016, Heska HT-5 control software, Phenix 1.19.2 |
| Data analysis | Graphpad Prism v9, Microsoft Excel v2016, Phenix 1.19.2, Chimera-X v1.5, Coot 0.8.9.2, Snapgene v6.2, Pymol v2.5.2 |

For manuscripts utilizing custom algorithms or software that are central to the research but not yet described in published literature, software must be made available to editors and reviewers. We strongly encourage code deposition in a community repository (e.g. GitHub). See the Nature Portfolio guidelines for submitting code & software for further information.

## Data

Policy information about availability of data

All manuscripts must include a data availability statement. This statement should provide the following information, where applicable:

- Accession codes, unique identifiers, or web links for publicly available datasets
- A description of any restrictions on data availability
- For clinical datasets or third party data, please ensure that the statement adheres to our policy

The structural data that supports these findings is available in the Protein Data Bank under accession numbers 8F8V (https://doi.org/10.2210/pdb8F8V/pdb), 8F8W (https://doi.org/10.2210/pdb8F8W/pdb), 8F8X (https://doi.org/10.2210/pdb8F8X/pdb), 3AVE (https://www.rcsb.org/structure/3ave), and 3GSK (https://www.rcsb.org/structure/3GSK).

## Human research participants

Policy information about studies involving human research participants and Sex and Gender in Research.

| | |
|---|---|
| Reporting on sex and gender | No human research participants. |
| Population characteristics | No human research participants. |
| Recruitment | No human research participants. |
| Ethics oversight | No human research participants. |

Note that full information on the approval of the study protocol must also be provided in the manuscript.

# Field-specific reporting

Please select the one below that is the best fit for your research. If you are not sure, read the appropriate sections before making your selection.

☒ Life sciences ☐ Behavioural & social sciences ☐ Ecological, evolutionary & environmental sciences

For a reference copy of the document with all sections, see nature.com/documents/nr-reporting-summary-flat.pdf

# Life sciences study design

All studies must disclose on these points even when the disclosure is negative.

| | |
|---|---|
| Sample size | No statistical method was used to predetermine sample size. On the basis of previous studies (Yamin et al., Biorxiv 2023) that determined experimental variation in survival following antibody-dependent enhancement of dengue infection, we performed power calculations and determined that at least n=6 animals per group is sufficient to detect differences among experimental groups. Sample sizes were limited by mouse colony size and breeding constraints. |
| Data exclusions | No data was excluded. |
| Replication | Structure of the X0-afucosylated IgG1 Fc complex was internally validated by 2 separate crystallization conditions in distinct space groups with an RMSD < 1.5Å for all atoms. For surface plasmon resonance studies in Figures 2 and 3, each mutant was tested across multiple serial dilutions, confirming the appropriate nanobody-antibody affinities. The study in Figure 5 is the result of two pooled experiments which yielded similar results. |
| Randomization | Group allocation in Fig. 5 was performed randomly and each mouse cage contained mice from each group, controlling for littermate differences. Other experiments do not contain groups, and thus do not require randomization. |
| Blinding | One of the two co-authors was blinded to groups before collection and analysis of the data in Fig. 5, preventing bias of data interpretation. Other experiments do not contain groups, and thus do not require blinding. |

# Reporting for specific materials, systems and methods

We require information from authors about some types of materials, experimental systems and methods used in many studies. Here, indicate whether each material, system or method listed is relevant to your study. If you are not sure if a list item applies to your research, read the appropriate section before selecting a response.

## Materials & experimental systems

| n/a | Involved in the study | |
|---|---|---|
| ☐ | ☒ | Antibodies |
| ☒ | ☐ | Eukaryotic cell lines |
| ☒ | ☐ | Palaeontology and archaeology |
| ☐ | ☒ | Animals and other organisms |
| ☒ | ☐ | Clinical data |
| ☒ | ☐ | Dual use research of concern |

## Methods

| n/a | Involved in the study | |
|---|---|---|
| ☒ | ☐ | ChIP-seq |
| ☒ | ☐ | Flow cytometry |
| ☒ | ☐ | MRI-based neuroimaging |

## Antibodies

| | |
|---|---|
| Antibodies used | Anti-DENV antibody clone C10 was used and produced in-house as detailed in the Methods section. |
| Validation | Antibodies were validated for target specificity as outlined in Yamin et al., Biorxiv 2022. |

## Animals and other research organisms

Policy information about studies involving animals; ARRIVE guidelines recommended for reporting animal research, and Sex and Gender in Research

| | |
|---|---|
| Laboratory animals | In vivo experiments were approved by The Rockefeller University Institutional Animal Care and Use Committee in compliance with federal laws and institutional guidelines. Mice were maintained at the Comparative Bioscience Center at the Rockefeller University at a controlled ambient temperature (20–25°C) and humidity (30–70%) environment with 12-h dark:light cycle. In all experiments, Ifnar1-/- FcγR-humanized mice (males and females; 4-5 weeks old) were used. See Yamin et al., Biorxiv 2022 for details on the mouse strain. |
| Wild animals | No wild animals were used. |
| Reporting on sex | Experiments in Fig. 5 used both sexes in all groups, indicating that sex was not a primary determinant of disease outcomes. |
| Field-collected samples | No field-collected samples were used. |
| Ethics oversight | All in vivo experiments were performed in compliance with federal laws and institutional guidelines and have been approved by the Rockefeller University Institutional Animal Care and Use Committee. Mice were bred and maintained at the Comparative Bioscience Center at the Rockefeller University. |

Note that full information on the approval of the study protocol must also be provided in the manuscript.
